# Follow‐Up of 3‐Year Luspatercept Treatment in a Transfused ß‐Thalassemia Patient. Bone Marrow: An Undervalued Iron Store

**DOI:** 10.1002/jha2.70193

**Published:** 2025-11-27

**Authors:** Matthias Bleeke, Claudia Niekrens, Soumya Datta, Regine Grosse, Filomena Longo, Isabel Molwitz, Roland Fischer

**Affiliations:** ^1^ Department of Pediatric Hematology and Oncology University Medical Center Hamburg‐Eppendorf Hamburg Germany; ^2^ Division of Pediatric and Adolescent Medicine Delme Klinikum Delmenhorst Delmenhorst Germany; ^3^ Department of Pediatric Hematology and Oncology Gemeinschaftsklinikum Mittelrhein Koblenz Germany; ^4^ Department of Thalassemia and Hemoglobinopathy University of Ferrara Cona‐Ferrara Italy; ^5^ Department of Diagnostic and Interventional Radiology University Medical Center Hamburg‐Eppendorf Hamburg Germany

## Abstract

**Introduction:**

Treatment with luspatercept may improve transfusion requirements in transfusion‐dependent thalassemia (TDT), but the improved erythroid maturation in the bone marrow influences body iron distribution.

**Case Report:**

We report on sequential organ iron measurements in a TDT patient under luspatercept treatment. Despite a decline in transfusion requirement and ferritin, we observed a redistribution of body iron stores from spleen and bone marrow to the liver, increasing liver iron concentration (LIC).

**Conclusion:**

Luspatercept treatment affects the informative value of ferritin and LIC in the assessment of total body iron stores, which should be considered in the management of iron chelation therapy.

## 1 Introduction

Patients with transfusion‐dependent β‐thalassemia (TDT) suffer from ineffective erythropoiesis due to defective or missing production of ß‐globin chains, resulting in a toxic imbalance of the α/ß‐globin ratio, and require lifelong regular blood transfusions. Blood transfusion leads to iron accumulation in various organs such as the liver, heart, pancreas, bone marrow, or pituitary gland and causes significant morbidity and mortality if not sufficiently treated [1]. Treatment with iron chelators such as deferoxamine, deferiprone (DFP), or deferasirox (DFX) is therefore mandatory in all transfusion‐dependent patients. Regular assessment of iron overload is needed to ensure adequate treatment and to avoid under‐ as well as over‐chelation. Although ferritin is usually inexpensive and readily available, variations in ferritin only partially reflect the changes in the hepatic and, therefore, total body iron (TBI) load [2]. Measurement of internal organ iron deposition by MRI is more reliable than ferritin alone, and repeated measurement of liver iron concentration (LIC) allows a more accurate estimation of the iron balance to ensure optimal iron chelation therapy [3, 4]. As curative treatment options such as allogeneic blood stem cell transplantation or gene therapy are not available to all patients with TDT, reduction of transfusion requirements through the use of disease‐modifying therapies is desirable. Luspatercept, a scavengermolecule of TGF‐β superfamily ligands, inhibits activation of activin receptors with subsequent blocking of Smad2/3 signaling pathways in erythropoietic progenitors [5]. Blocking Smad2/3 signaling results in increased erythroid maturation with improvement of anemia in diseases with ineffective erythropoiesis [6]. Luspatercept has shown clinical efficacy in TDT and NTDT patients [7].

## 2 Case Presentation

Here we report the case of a male patient with TDT (genotype ß^0^/ß^0^) on a regular blood transfusion and iron chelation program treated with luspatercept for 39 months. The initially 22‐year‐old patient attended our hematology unit from 1999 to 2025 for consultation and annual iron assessment by liver biosusceptometry and later by MRI chemical shift relaxometry (MRI‐CSR) at 1.5 Tesla (T) and 3 T, respectively [8]. Iron concentration (R2*) and fat fraction (FF) were simultaneously assessed by MRI‐CSR, as well as organ volumes from consecutive axial slices by 3D mDixon MRI. Relaxation rates (R2*) and iron concentrations were analyzed from one representative liver (LIC) and spleen (SIC) slice. Bone marrow R2* was determined from the same image slice in the center of the 12th thoracic vertebra (VBM). Minor iron amounts (R2*) were assessed in pancreas tail (PIC) and septal heart (CIC). All MRI‐R2* values at 3.0 T were converted to 1.5 T [9]. Since 2010, DFP and DFX have been used in combination for iron chelation treatment on a daily basis. Although the patient had a good adherence to iron chelation treatment, LIC and ferritin were persistently high, with LIC at about 18 mg/g_dry weight_, ferritin levels at 4000 µg/L, normal liver volume (1400 mL), and moderate splenomegaly (450 mL). However, the chelation doses could not be increased due to adverse effects (leukopenia, creatinine increase).

**FIGURE 1:**
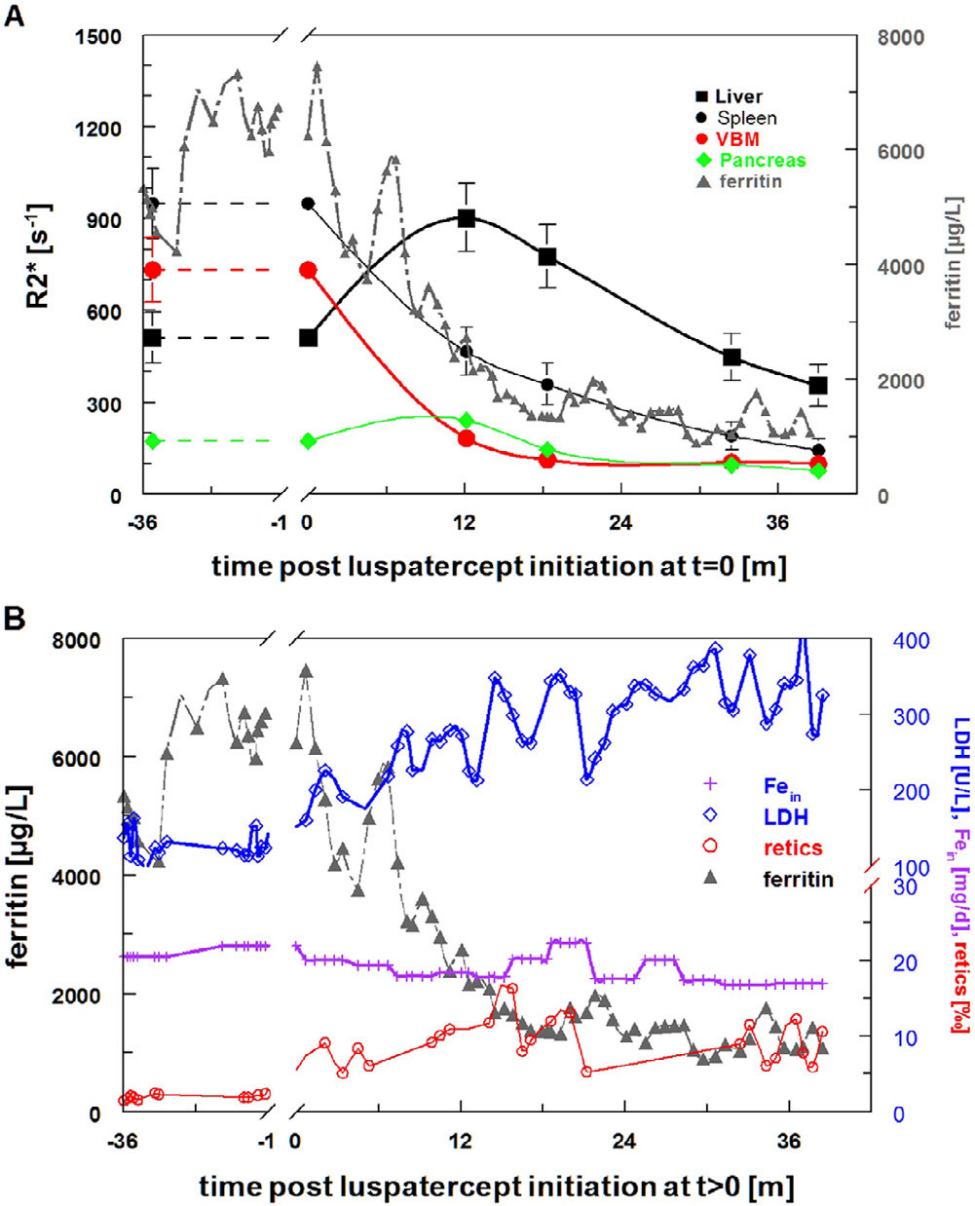
(A) Iron parameters in the course of luspatercept treatment. Left axis: MRI‐R2* relaxation for liver, spleen, pancreas (tail), and vertebral bone marrow; right axis: ferritin. R2* rates before luspatercept treatment were extrapolated to baseline (dashed lines). (B) Hematological parameters. Left axis: ferritin for direct comparison; right axis: lactate dehydrogenase (LDH), daily iron input rate (Fe_in_), and reticulocytes (retics).

Before initiation of luspatercept treatment (−33.6 months, Figure 1A), liver R2* was 510/s (15.9 mg/g_d.w._), spleen R2* 949/s (4.2 mg/g_d.w._), VBM‐R2* 733/s, and pancreatic R2* 174/s. The mean daily iron input rate from RBC transfusions was 20.5 mg/d before luspatercept treatment. A combined DFP and DFX chelation regimen was applied with twice daily dosing, adding to total dose rates of 51.7 and 15.5 mg/kg/d, respectively. Since this treatment regimen was not changed during the next 30 months, and indirect signs of iron load (ferritin, pre‐transfusional Hb, etc.) were constant, we could extrapolate the above organ iron concentrations (or R2*) to baseline.

After starting luspatercept treatment at the age of 45 years with 1.0 mg/kg every 3 weeks, ferritin levels rapidly decreased while LDH increased, as well as a rising number of reticulocytes emerged from the erythroid (Figure 1B). VBM‐R2* decreased from 733/s before luspatercept to 183/s after 1 year of luspatercept treatment and converged towards normal values of 57–119/s after 3 years of treatment (Figure 1A). In parallel, spleen iron followed a similar trajectory, but did not reach normal R2* levels of 18–54/s. However, liver R2* increased to 902/s (LIC = 23.4 mg/g_d.w._) during the first year of luspatercept treatment, despite a 16% reduction in RBC iron influx and unaltered iron chelation regimen. Because of increased LIC, iron chelation doses were adjusted, resulting in a decrease in LIC reaching a sub‐baseline value (before luspatercept initiation) of 12.4 mg/g_d.w._ (Table 1). Pancreatic R2* took a similar course with increased pancreatic R2* after 12 months of luspatercept treatment and a decrease after adaptation of iron chelation. Cardiac iron was in the range of R2* = 31–35/s (normal < 40/s) during to whole observation period. The MRI examinations revealed no evidence of extramedullary erythropoiesis before or during luspatercept treatment.

**TABLE 1:** Quarterly clinical parameters (mean ± SD) and calculated parameters at ‐34, baseline (‐1 m), 12, 18, 33, and after 39 months post initiation of luspatercept treatment.

**Parameter**	**−34 m**	**Baseline**	**12 m**	**18 m**	**33 m**	**39 m**
Hb (g/dL)	10.2 ± 0.2	9.9 ± 1.0	10.5 ± 0.5	9.5 ± 1.1	9.7 ± 0.8	10.6 ± 1.2
Fe_in_(RBC) (mg/d)	20.5	21.9	18.4	20.2	20.1	16.9
Ftn (µg/L)	4988 ± 286	6464 ± 292	2851 ± 388	1506 ± 163	1052 ± 141	1283 ± 246
LIC (mg/g_d.w._)^a^	15.9 ± 2.6	15.9^c^	23.4 ± 2.9	21.1 ± 2.8	14.6 ± 2.5	12.4 ± 2.3
Liver volume (mL)^b^	1307	1307	1377	1351	1339	1399
SIC (mg/g_d.w._)^a^	24.2 ± 2.9	24.2^c^	14.9 ± 2.5	12.5 ± 2.4	8.1 ± 1.9	6.6 ± 1.7
Spleen volume (mL)^b^	509	509	499	455	457	485
LDH (U/L)	135 ± 25	133 ± 18	271 ± 7	288 ± 30	346 ± 42	329 ± 49
Bilirubin‐tot. (mg/dL)	1.0 ± 0.2	0.7 ± 0.2	1.6 ± 0.2	1.7 ± 0.1	1.9 ± 0.2	1.7 ± 0.1
AST (< 50 U/L)	55 ± 95	58 ± 10	95 ± 18	68 ± 5	71 ± 11	73 ± 13
ALT (< 50 U/L)	29 ± 6	32 ± 12	35 ± 1	35 ± 9	28 ± 2	20 ± 10
DFP (mg/kg/d)	51.7	51.7	63.6	72.7	71.4	69.0
DFX (mg/kg/d)	15.5	15.5	13.7	13.1	12.2	12.4

Abbreviations: ALT, alanine aminotransferase; AST, aspartate aminotransferase; DFP, deferriprone; DFX, deferasirox; Fein(RBC), iron influx rate calculated from transfused packed red blood cells; Ftn, ferritin; LDH, lactate dehydrogenase; LIC, liver iron concentration (µg/g_liver_); SIC, spleen iron concentration (µg/g_spleen_).

^a^ Based on data at these time points, see Figure 1A.

^b^ Uncertainties of liver and spleen volume determinations are 75 and 25 mL, respectively.

^c^ Extrapolated from measurements at −34 months.

Treatment with luspatercept has the potential to significantly reduce the transfusion requirements in thalassemia patients with ß^0^/ß^0^ genotype [10]. The patient under study achieved a 16% reduction in transfusion requirement after 1 year of luspatercept treatment with a significant decrease in ferritin values (Table 1). In contrast, liver iron levels significantly increased over the same period. The observation that luspatercept treatment leads to a decrease in ferritin while the LIC remains stable has already been observed in patients in the BELIEVE trials of luspatercept in TDT.

In summary (Table 1), the following mean quarterly clinical observables were obtained at baseline (−33.6 months), after 1 year, 1.5 years, and finally after more than 3 years of luspatercept treatment together with calculated parameters. The slightly higher activity of AST in patients under luspatercept is probably the result of numerous defective red blood cells, especially since this correlates with increasing LDH, bilirubin, and reticulocytes.

In a subgroup analysis of spleen iron levels in patients in the BELIVE and BEYOND trials of luspatercept in TDT and NTDT, spleen iron correlated well with liver iron prior to luspatercept treatment, but unlike LIC, spleen iron levels decreased significantly with luspatercept, leading to a marked decrease in the correlation between iron levels in these two organs [11].

This effect of “deironing the spleen” by luspatercept [12] could also be demonstrated in our patient with spleen relaxation rates R2* dropping significantly from 949/s ( = 24.2 mg/g_spl_) before luspatercept to 466, 359, and 191/s after 12, 18, 33, and 39 months of luspatercept treatment, respectively. Bone marrow iron concentration also decreased, but more markedly than in the spleen, reflecting the increased utilization of iron stored in reticuloendothelial cells in response to erythroid maturation treatment. In addition, there was a redistribution of iron stores to the liver, leading to an increase in LIC. Redistribution of iron from macrophages in bone marrow and spleen to hepatocytes has previously been observed in patients treated with luspatercept. In this model, increased erythroid maturation causes a decrease in hepatic hepcidin release as a result of increased erythroferrone. This enables iron to be released from macrophages, which is associated with a decrease in serum ferritin. This released iron, as well as iron from increased hemolysis of thalassemic cells, is taken up by the liver, increasing LIC [13].

**FIGURE 2:**
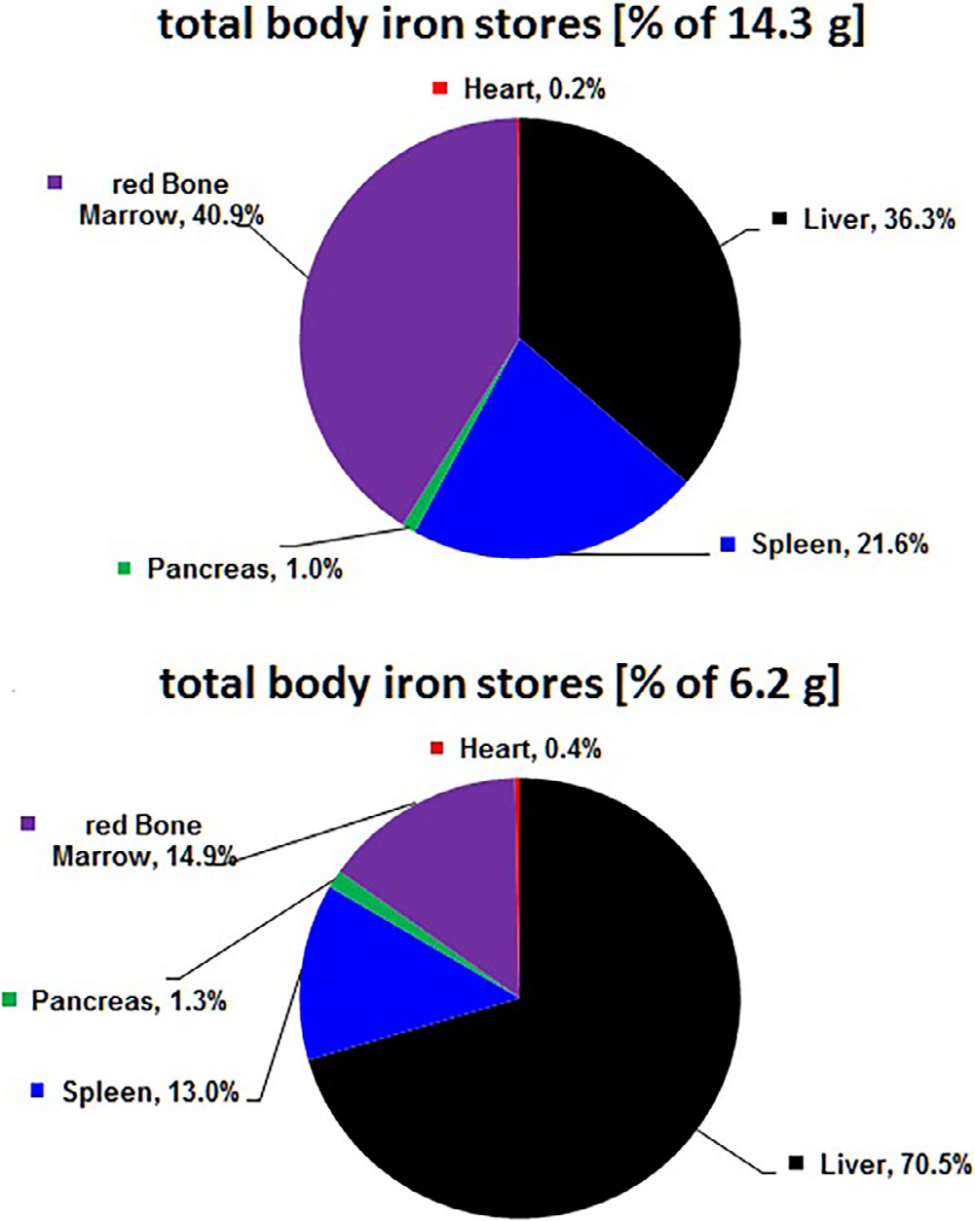
Redistribution of total body iron stores (TBI) before (upper panel) and after 39 months (lower panel) of luspatercept treatment, showing organ iron amounts as percentages of TBI. Liver iron of 5.2 g at baseline (upper panel) only decreased to 4.3 g post 39 months (lower panel), in contrast to 3.1 versus 0.8 g in the spleen. While the reduction of iron in red bone marrow from 5.8 to 0.9 g reflected the release of iron due to the improving erythroid maturation.

Mobilizable TBI stores were derived from iron concentrations in different organs multiplied by their respective volumes or masses. Iron concentrations were calculated from R2* using calibrations by liver biosusceptometry [9] or more directly by MR cardio susceptometry [14]. Beyond liver and spleen, these calibrations were also applied to the pancreas and the VBM. Especially, no calibrations of VBM‐R2* by chemically determined bone marrow iron are currently available, and using normal bone marrow mass relations from ICRP might be debatable in patients with TDT. Heart mass was estimated at 4 g/kg body weight, and red (active) marrow mass was assumed to be 1.6% of body weight [15]. With these assumptions, TBI was calculated as the sum of iron in all five organs before and after 39 months of luspatercept treatment. As shown in Figure 2, a significant redistribution of iron takes place under luspatercept treatment. In agreement with Garbowski et al. [12], released iron from the macrophages in the bone marrow and spleen is taken up by the liver (and pancreas), in part as non‐transferrin bound iron, and thereby removed in the chelation process.

## 3 Conclusion

While the use of luspatercept may reduce transfusion requirements in patients with TDT, the redistribution of iron caused by increased erythropoiesis in the bone marrow directly affects the informative value of commonly used parameters of iron overload as ferritin and LIC. In patients with TDT treated with an erythroid maturation agent, it is not only the liver and spleen iron that reflects TBI, but to a greater extent, the active (red) bone marrow. It is important to be aware of these changes in order to make appropriate adjustments to iron chelation in patients with TDT receiving luspatercept.

## Author Contributions

M.B. and R.F. wrote the manuscript. R.F. collected and analyzed the data. C.N., D.S., R.G., F.L., and I.M. collected data and contributed to manuscript preparation.

## Funding

The authors have nothing to report.

## Ethics Statement

The authors have nothing to report.

## Consent

The patient reported here gave full consent for the case presentation.

## Conflicts of Interest

The authors declare no conflicts of interest.

## Data Availability

Due to the sensitive nature of the clinical data, access is restricted. Data may be available upon reasonable request and with permission of the patient.
